# Pharmacogenetics of follicle‐stimulating hormone action in the male

**DOI:** 10.1111/andr.70053

**Published:** 2025-04-30

**Authors:** Andrea Graziani, Giuseppe Grande, Raffele Scafa, Riccardo Selice, Andrea Garolla, Maria Santa Rocca, Cinzia Vinanzi, Alberto Ferlin

**Affiliations:** ^1^ Department of Medicine University of Padova Padova Italy; ^2^ Department of Systems Medicine, Unit of Andrology and Reproductive Medicine University Hospital of Padova Padova Italy

**Keywords:** FSH, FSHB, FSHR, genetics, male factor infertility, polymorphisms

## Abstract

Male factor infertility (MFI) is involved in half of the cases of couple infertility. The follicle‐stimulating hormone (FSH) therapy is considered efficient to improve semen parameters and pregnancy rate in patients with idiopathic MFI, following the lesson learned from hypogonadotropic hypogonadism. However, while in patients with hypogonadotropic hypogonadism FSH therapy, in combination with human chorionic gonadotropin (hCG), is a well‐established treatment, in patients with MFI the effects of the FSH therapy are variable and unpredictable. The FSH therapy in MFI should be a personalized treatment, tailored on the characteristics of the male patient and the couple. The pivotal aspect is the accurate identification of patients who might benefit from such treatment (responders) from those who might not (nonresponders). To date, selection of patients to be treated is based on history, physical examination, semen analysis, and hormonal assessment. However, these parameters cannot adequately identify a priori responder patients. Furthermore, tailored management should include pharmacological adaptation (dosage and duration of the therapy), as happens during ovarian hyperstimulation in assisted reproductive technologies. In a fully personalized therapy, pharmacogenetic factors must be considered. In this paper, we describe the evidence dealing with the pharmacogenetics of the FSH therapy in MFI, presenting the physiological and physiopathological basis and the pharmacogenetics studies dealing with effects of polymorphisms in the beta‐subunit of FSH (*FSHB*) and the FSH receptor (*FSHR*) gene. According to the evidence so far available, genetic evaluation of *FSHB* and *FSHR* is recommended only for research purposes, since the data are not conclusive and even contrasting. Furthermore, the evidence so far is derived from quite small studies with different endpoints considered and relatively few cases. Better studies that consider the combined effect of several *FSHB* and *FSHR* gene polymorphisms, together with clinical, biochemical, seminal and testicular cytology, are necessary to develop an algorithm that might predict the response to the FSH treatment.

## 1 INTRODUCTION

Infertility, defined as being unable to conceive after 12 months of regular and unprotected sexual intercourse, is nowadays a well‐recognized clinical (and social) condition, affecting about 10%–15% of couples worldwide.^1^, ^2^, ^3^ Infertility should not be viewed as a matter solely related to the woman or the man, being indeed always a problem involving the couple. This approach means that the diagnostic and therapeutic process should be performed in parallel for both partners.^4^, ^5^

Male factor infertility (MFI) is involved, alone or in combination with a female factor, in about half of the overall cases of couple infertility.^6^ MFI harbors a great number of etiologies, including genetic mutations, lifestyle factors, infections, testicular alterations, varicocoele, systemic diseases and drugs.^7^, ^8^, ^9^, ^10^ Indeed, MFI represents the perfect example of a complex disease with a substantial genetic basis, being genetic alterations involved in at least 10%–15% of the cases.^11^, ^12^ Therefore, the genetic investigation represents an essential and useful diagnostic tool, mainly in azoospermic and severely oligozoospermic men.^6^, ^11^, ^12^, ^13^, ^14^ Advances in the understanding of MFI and its genetic components will—in a near future—improve the clinical application of genetic evaluations to clarify the causes of several other forms of reduced fertility in males, beyond the evaluation of karyotype anomalies and Y chromosome microdeletions.

Idiopathic MFI, defined in patients without evidence of a defined cause or clinical condition associated with infertility and semen alterations, has been reported in up to 30%–50% of the cases of MFI, although important differences exist among the different studies, due to the different grades of diagnostic performed in the protocols.^6^, ^15^ Although it is pretty evident that the term “idiopathic” should be used only after a precise and complete diagnostic process, guidelines in fact do not agree on the correct management of MFI, which unfortunately, in practice is often limited to semen analysis.^5^ Indeed, a valid clinical andrological diagnostic workup, along with subsequent treatments, cannot rely only on semen analysis alone, therefore requiring a complete diagnostic workflow.^16^ Although these premises, the high proportion of idiopathic MFI, even after a comprehensive diagnostic workup, suggests that the mechanisms regulating spermatogenesis and sperm function remain largely unknown.

Several hormonal approaches have been developed to improve sperm concentration in patients with idiopathic MFI, including the use of follicle‐stimulating hormone (FSH). Although attempts have been made to develop an algorithm predicting the response to the FSH therapy,^17^ it is evident that we are still far from being able to clearly identify those infertile patients that will respond to the FSH treatment. Indeed, several data have been published underlining the importance of different clinical, laboratory, and genetic parameters in the prediction of the response to the FSH treatment.^18^

## 2 PHARMACOGENETIC APPROACH TO FOLLICLE‐STIMULATING HORMONE THERAPY IN MALE INFERTILITY

For qualitative and quantitative normal spermatogenesis, an intact hypothalamic–pituitary–gonadal axis is essential. Gonadotropin releasing hormone (GnRH) is released by the hypothalamus. In turn, GnRH stimulates the pituitary to secrete luteinizing hormone (LH) and FSH. The pituitary gland therefore produces FSH, a dimeric glycoprotein that targets both male and female gonadal cells. The molecule shares structural similarities to LH, which works in conjunction with FSH to regulate the reproductive function, steroidogenesis, cell metabolism, and growth through certain G protein‐coupled receptors (GPCRs).^19^

The spermatogenetic process occurs within testicular seminiferous tubules and normal FSH concentrations are related to adequate spermatogonial quantity. On the other hand, endogenous FSH concentrations increase when spermatogonia are absent or significantly reduced.^18^

The primary—and main—role of FSH is to increase the number of sperm cells in synergy with intratesticular testosterone (ITT). FSH induces Sertoli cell proliferation during fetal life, then mini‐puberty and puberty (for the induction of spermatogenesis), while LH stimulates steroidogenesis in Leydig cells to produce androgens.^20^ In adult males, FSH acts through its receptor up to the secondary spermatocyte stage, promoting meiosis entry and limiting overall germ cell apoptosis, while ITT is responsible for the later spermatogenesis stages, comprising spermiogenesis.^21^, ^22^ Therefore, FSH and ITT regulate spermatogenesis in a synergistic manner.^23^

When the first gonadotropic substance was isolated from human pituitary glands in the 1960s, FSH was first proposed as infertility therapy. Several biosimilar and recombinant FSH molecules are now available, in addition to extremely pure urinary FSH solutions.^24^ Although some differences in the action on Sertoli cells of the different gonadotropins have been reported in in vitro studies,^25^ the clinical effects of recombinant and purified FSH (biosimilars and originators) are overall similar, being well tolerated and safe.

In the context of MFI, the FSH therapy is mainly used in two conditions: hypogonadotropic hypogonadism and idiopathic MFI.^6^ In patients with hypogonadotropic hypogonadism, the FSH therapy is well‐established, often in combination with the human chorionic gonadotropin (hCG) therapy. This treatment restores spermatogenesis in up to 90% of such patients, with spontaneous or assisted pregnancy rates observed in up to 65% of the patients.^6^, ^26^, ^27^ In patients with idiopathic MFI, the FSH treatment is suggested, according to most recent guidelines dealing with MFI, to improve sperm quantity, quality, and pregnancy rate when altered semen parameters are present alongside normal FSH concentrations, without any signs of obstruction in the seminal tract.^6^ However, the number needed to treat, based on combined results, ranges between 10 and 18, indicating that more than 15 men with MFI must be treated to achieve at least one pregnancy.^28^

The FSH therapy in MFI is, therefore, a personalized treatment which must be tailored to characteristics of the male patient and the whole couple.^29^ The main objectives of this treatment, defined as an empirical treatment of MFI^4^ are (i) to restore, when possible, natural male and couple fertility; (ii) to allow access to medically assisted reproduction (MAR); (iii) to allow a gradual approach to MAR procedures; and (iv) to improve pregnancy outcomes from MAR.

In clinical practice, only in Italy FSH is available (and its cost is covered by the National Health System) for men with infertility, otherwise hypogonadotropic hypogonadism. Despite this, a recent Italian survey reported that only 55% of patients eligible for FSH treatment under national rules were effectively treated with such therapy.^30^

Of note, no side effects or adverse events have been so far reported as a consequence of the FSH treatment in males, differently from women in which an excess of FSH might result in ovarian hyperstimulation syndrome. Furthermore, FSH‐secreting tumors in females lead to such syndrome, whereas in males no other effects than increasing testis size has been observed. Possible extragonadal effects of FSH are a very much debated issue and, for the time being, should not be a concern.^4^, ^18^, ^30^, ^31^

As in most cases of personalized treatment, the pivotal aspect of the FSH therapy is the accurate identification of patients who might benefit from such treatment. In fact, the response to the FSH treatment is often variable and unpredictable, probably because the factors influencing the response to FSH are not well clarified yet.^32^ In addition to semen evaluation, biochemical analysis and ultrasound assessment, a crucial factor is the ability to forecast an a priori identification of patients who will respond positively to the FSH therapy.^18^ Moreover, another important aspect to study is the pharmacological adaptation (i.e., dosage, duration, interval, …) tailored to the patient's characteristics, similar to what happens during ovarian stimulation for MAR. Besides genetic considerations, it is possible that some patients may need higher FSH dosage and/or longer therapy duration, while others might also respond to a lower FSH dosage. Indeed, it has to be noted that the FSH treatment in men with hypogonadotropic hypogonadism is a replacement and etiologic therapy, whereas in the context of MFI it is a stimulatory and empirical therapy.^4^

In this context, several polymorphisms within the *FSHB* and *FSHR* genes have been studied in clinical and experimental studies, as possible determinants of the physiological FSH plasma concentrations and the variable response to the FSH treatment. Notably, there are only few in vitro studies available dealing with the effect of *FSHB* and *FSHR* polymorphisms on FSH action in the male. This is mainly because appropriate read‐out systems for studying the FSH function do not exist and there are neither human gonadotropic nor Sertoli cell lines available.^33^

## 3 POLYMORPHISMS OF *FSHB* GENE

FSH is composed of a subunit that is shared with other glycoprotein hormones and a specific β‐subunit coded by the *FSHB* gene, which consists of three exons and is located on chromosome 11p21.^34^ The rate of transcription of FSHB regulates the quantity of FSH secreted.^35^, ^36^

*FSHB* gene has hundreds of polymorphisms, but to date the only clinically relevant is the *FSHB* c.‐211G > T polymorphism (rs10835638), which is located within the promoter of the gene, in the 5′ untranslated region, within an evolutionary conserved element.^33^ This polymorphism appears to be epidemiologically relevant only in Europe and USA, with 20%–25% of individuals carrying at least one T allele.^37^ This *FSHB* promoter polymorphism falls within a binding element for the LHX3 homeodomain transcription factor, which can influence gene transcription.^38^

According to the evidence so far obtained, the wild‐type promoter variant—carrying the G‐allele—has twice the activity compared to the “TT homozygous” variant. In other words, the relative activity of the *FSHB* promoter carrying the T allele is only half the activity of the wild‐type promoter variant with the G allele.^39^ Indeed, compared with the wild‐type homozygotes (GG), both the heterozygotes (GT) and the homozygotes (TT) for the alternative allele have reduced transcriptional activity and lower concentrations of plasma FSH, 15.7% and 40%, respectively.^36^, ^38^ Reduced FSH concentrations in patients harboring the T allele have been reported even in patients with Klinefelter syndrome.^40^ Other evidence found that homozygous carriers of the T allele represent only 1.5% of men with normal semen parameters but have serum FSH concentrations which are about 25% lower than homozygous major allele carriers (G).^37^

In a clinical setting, it was then reported that *FSHB* c.‐211G > T TT homozygous polymorphism was associated with lower total sperm count (TSC), lower testicular volume, lower serum Inhibin B, low serum testosterone, higher LH concentrations, lower serum FSH concentrations and decrease in FSH/LH ratio.^41^, ^42^, ^43^, ^44^ Therefore, patients bearing the T allele represent a sort of isolated FSH deficiency and cannot increase serum FSH concentrations to maintain spermatogenesis in cases of reduced testicular function. Therefore, these patients, even when affected by primary testicular damage (i.e., notoriously a condition characterized by elevated FSH plasma levels), might have low/normal FSH plasma concentrations.

A previous cross‐sectional study from our group reported that patients with MFI with *FSHB* c.‐211G > T TT homozygous variant respond better to the FSH therapy, showing a higher increase in spermatogenesis compared to carriers of the other genotypes. In fact, TT homozygotes showed a more pronounced increase in semen parameters, making them more likely than GT heterozygotes and GG homozygotes to become normozoospermic following the FSH therapy.^41^ Moreover, we recently reported that evaluation of such polymorphism might be useful—in some cases—even in conditions usually associated with higher FSH concentrations.^45^ Given this evidence, some studies advocate the implementation of *FSHB* genotyping as a routine diagnostic evaluation in selected patients with MFI.^46^

However, conflicting data regarding this polymorphism are present in the literature. A 2016 study by Simoni et al.,^47^ evaluating sperm DNA fragmentation index (DFI) improvement after the FSH therapy, reported that a significant improvement in such parameters was found only in carriers of the combination of a polymorphism in receptor of FSH (*FSHR* p.N680S N homozygous—see also above) and *FSHB* G homozygous genotypes. Moreover, when considering only the *FSHB* ‐211G > T genotype alone, irrespective of the *FSHR* genotype, DFI was not different between *FSHB* ‐211G > T homozygous G and *FSHB* ‐211G > T homozygous T and heterozygous G/T genotypes together, although brighter DFI decreased significantly only in the homozygous G group.

Indeed, a recent study reported that *FSHB* c.‐211G > T G homozygous was more frequently observed in patients with altered semen analysis—oligo‐astheno‐teratozoospermia—among 109 infertile men compared to fertile controls.^48^ Other authors found, in a cross‐sectional study of 2020 Danish men, that *FSHB* c.‐211G > T variant was associated with lower FSH concentrations and smaller testicular volume, but did not find significant correlation with other semen parameters, aside from a reduced number of morphologically normal spermatozoa in heterozygous carriers.^49^ In addition, a study by Casamonti et al.^50^ found no clear‐cut effect of genotype (*FSHB* c.‐211 TT vs. GT vs. GG) in predicting response to the FSH treatment, with treatment outcomes appearing to be independent of the *FSHB* polymorphism. Finally, another recent study,^51^ evaluating 1075 azoospermic men undergoing testicular sperm extraction (TESE), showed that *FSHB* c.‐211G > T TT homozygosity was associated with a lower likelihood of sperm retrieval.

Regarding the risk of transmission, no data have been published so far about the impact of paternal *FSHB* gene polymorphisms on the reproductive health of the offspring. Thus, genetic evaluation of the female partner should be performed in order to assess the risk of TT homozygosity or GT heterozygosity.^45^

## 4 POLYMORPHISMS OF *FSHR* GENE

The FSHR belongs to the GPCRs family, which is involved in several signaling pathways.^52^ In men, FSHR is located mainly on Sertoli cells, which are pivotal for both the quantity and the quality of normal spermatogenesis.^53^ The *FSHR* gene, located on chromosome 2p21, consists of 10 exons.^33^ The two main polymorphisms of *FSHR* are Thr307Ala (c.919A > G; rs6165) and Asn680Ser (c.2039A > G; rs6166), which are located in exon 10.^33^ These *FSHR* polymorphisms have been the most studied as possible pharmacogenetic markers for the efficacy of FSH treatment.^18^ Another polymorphism, the rs1394205 (c.‐29G > A), is located in the promoter region of the *FSHR* gene^33^ and has been studied less extensively.

When dealing with the two main polymorphisms, it has to be said that rs6165 and rs6166 are in strong linkage disequilibrium, therefore it is sufficient in most cases to analyze one of them. They are located in the large extracellular portion of the FSHR, likely contributing to ligand binding.^28^ In particular, *FSHR* c.2039A > G (N680S) is associated with receptor sensitivity: the variant Asn/Asn (A homozygous) is associated with the highest sensitivity and the variant Ser/Ser (G homozygous) with lowest sensitivity.^37^, ^54^ This polymorphism has been associated with testicular volume in men^53^ and, in particular, a significant association between the FSHR Ser680 allele (G) and lower total testes volume was reported. Additionally, it was found that men with the homozygous G variant (Ser/Ser) exhibited higher FSH serum concentrations compared to those with homozygous A (Asn/Asn) or heterozygous A/G (Asn/Ser) genotypes.^44^ Nevertheless, conflicting evidence and data exists regarding this polymorphism, such as the lack of difference in FSH concentrations, reproductive function, and distribution in fertile and infertile patients.^55^

Conflicting evidence is reported even when dealing with the clinical impact of such polymorphisms according to the FSH therapy. A pilot study involving 70 oligozoospermic patients^56^ showed that patients carrying Ser‐allele of the FSHR Asn680Ser polymorphism (therefore, FSHR p.N680S S homozygous or heterozygous patients) had a significant increase in TSC, sperm concentration, motility, and percentage of normal morphology forms after therapy with FSH. In contrast, other authors^47^ found that only FSHR p.N680S N homozygosity was associated with a decrease in the sperm DFI after FSH treatment, indicating that FSHR p.N680S N homozygous responded to such therapy. Another study,^50^ analyzing FSH therapy in about 40 patients with MFI, reported that the responsiveness to treatment resulted independent from FSHR/FSHβ polymorphisms (*FSH*β −211G > T, *FSHR* 2039A > G and FSHR −29G > A genotypes). A more recent prospective longitudinal study, evaluating patients with MFI before and after FSH therapy, found that the FSHR c.2039 genotypes and allelic distribution did not differ between responders and nonresponders.^57^

The other common polymorphism of FSH, almost equally common in Caucasian population as the previous one, is the rs6165 (c.919A > G, p.T307A). This polymorphism (A allele) has been associated with lower receptor activation.^37^ One study reported higher sperm concentrations in patients harboring the p.Thr307Ala variant compared to the p.Thr307Thr and p.Ala307Ala variants. Moreover, the same study found that patients with p.Ala307Ala genotype had lower fertilization rate (medically assisted reproduction outcomes) than patients with the p.Thr307Thr genotype.^58^ Other authors found an association between the said polymorphism and the presence of MFI.^59^

The least studied *FSHR* polymorphism is rs1394205 (–29G > A), located on the promoter of *FSHR* gene, which has been shown to reduce the transcriptional activity of *FSHR* in vitro^60^ and might, alone or in combination with other polymorphisms, modulate FSH concentrations in men, with G allele associated with higher receptor expression and lower FSH concentrations and A allele associated with lower receptor expression and higher FSH concentrations.^43^ To the best of our knowledge, only two studies have evaluated the impact of this least studied polymorphism. The first study^50^ reported that the responsiveness to FSH treatment in 39 patients with MFI was independent from *FSHR* c. ‐29G > A polymorphism and the second study^57^ found—in 23 patients undergoing the FSH therapy and genetic evaluation—that *FSHR* c. ‐29G > A genotypes and allelic distributions did not differ among responders and non‐responders to the FSH therapy.

In addition, in a meta‐analysis it was investigated the effect on MFI of the three polymorphisms of *FSHR* and the polymorphism of *FSHB* and it was found that the combination of three polymorphisms of *FSHR (FSHR* c.‐29G, c.919A, c.2039A) seemed protective against MFI than either one alone.^59^

## 5 FUTURE PERSPECTIVES AND CONCLUSION

**FIGURE 1:**
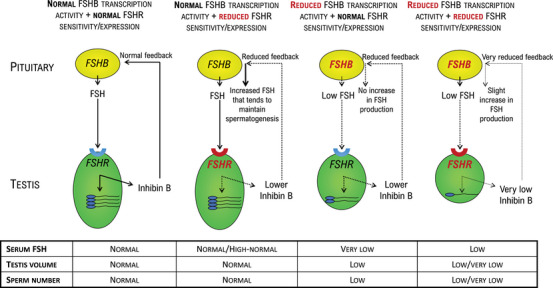
Physiological and pathophysiological effects of polymorphisms in the beta‐subunit of FSH (*FSHB*) and the FSH receptor (*FSHR*) gene. The standard condition is associated with normal FSH concentrations, normal testicular volume and normal total sperm count. In the presence of reduced FSHR sensitivity or expression (due to *FSHR* gene polymorphisms) with normal *FSHB* transcription, patients typically have normal/high‐to‐normal FSH concentrations, and normal testicular volume and normal total sperm count sustained by increased FSH plasma concentrations. In the presence of reduced *FSHB* transcription with normal FSHR sensitivity and expression, patients present low FSH concentrations, decreased testicular volume and a lower sperm number, being unable to increase FSH production despite lower inhibiting feedback. Finally, when both *FSHB* transcription and FSHR sensitivity or expression are reduced, patients have low FSH concentrations, low/very low testicular volume and low/very low total sperm count. FSH, follicle‐stimulating hormone.

It is evident that polymorphisms in *FSHB* and *FSHR* genes have important physiologic and pathophysiologic implications in the testicular function (Figure 1). However, according to the evidence described here and summarized in Table 1, it is clear that no definitive data can be used to support or not a pharmacogenetic approach to the FSH therapy in MFI.

**TABLE 1:** Pharmacogenetics studies dealing with polymorphisms in the beta‐subunit of FSH (FSHB) and the FSH receptor (FSHR) gene (modified from Schubert et al.^29^).

Study	Study type	Population size	SNP selection	Inclusion criteria for FSH treatment	FSH treatment utilized	Primary end‐point	Results
Ferlin et al.^30^	Cross‐sectional and prospective study, monocentric	67	*FSHB* c.‐211 G > T polymorphism **(GG, GT, TT)**	FSH ≤ 8 + TSC < 40 × 10^6^ Mil/ejac or azoospermia	rh‐FSH 150IU three times a week, for 3 months	TSC	GG, GT: ↑ TT: ↑↑
Casamonti et al.^31^	Prospective study, monocentric	40	*FSHB* c.‐211 G > T polymorphisms (**GG, GT, TT**) *FSHR* p.N680S polymorphisms (**S/S, S/N** and **N/N**) c.‐29 G/A polymorphisms (**GG, GA, AA**)	FSH < 8 + oligo‐/astheno‐/terato‐ zoospermia (OAT)	hp‐FSH 75IU every other day, for 3 months	Sperm hyaluronic acid binding capacity	*FSHB* c.‐211 G > T GG, GT, TT: ↑ *FSHR* p.N680S S/S, S/N and N/N: ↑ *FSHR* c.‐29 G/A GG, GA, AA: ↑
Selice et al.^32^	Prospective RCT, monocentric	70/35	*FSHR* p.T307A p.N680S polymorphisms (**TN/TN, TN/AS, AS/AS**)	FSH 1–8, sperm conc. < 20 × 10^6^/mL, testicular citology of hyposmermatogenesis without maturative arrest	rh‐FSH 150 IU three times a week, for 3 months vs. no treatment	TSC	TN/TN:—TN/AS: ↑ AS/AS: ↑
Simoni et al.^33^	Prospective, longitudinal, open‐label, multicentric	55	*FSHB* c. ‐211 G > T polymorphisms (**GG, GT, TT**) *FSHR* p.N680S polymorphisms (**S/S, N/N**)	FSH < 8 DFI > 15% normo‐ or oligo‐zoospermia	rh‐FSH 150 IU every other day, for 3 months	DFI	*FSHR* p.N680S N/N: ↓ *FSHR* p.N680S N/N and *FSHB* c.‐211 G > T GG: ↓
Mongioì et al.^34^	Prospective, monocentric	23	*FSHR* p.N680S polymorphisms (**S/S, S/N** and **N/N**) c.‐29 G/A polymorphisms (**GG, GA/AA**)	Idiopathic infertility	hp‐FSH 150 IU three times a week, for 4 months	Conventional, biofunctional sperm parameter and oxidative stress indicators	No effects

Abbreviations: DFI, Sperm DNA fragmentation index; FSH, follicle‐stimulating hormone; hp‐FSH, highly‐purified follicle‐stimulating hormone; RCT, randomized controled trial; rh‐FSH, recombinant human follicle‐stimulating hormone; SNP, nucleotide polymorphism; TSC, total sperm count.

In fact, the studies so far published and exposed are different ones each, with difficulties in proper comparison. In detail, they often differ because of the type of study conducted, they are often monocentric, they frequently lacked a control group and evaluation of female partners and pregnancy rate. Moreover, there is often a difference in the endpoint definitions, the number and types (inclusion criteria) of patients evaluated—mainly dealing with specific inclusion criteria for sperm parameters, the therapy administered (dosage and length), as well as the type of FSH administered. Thus, no reliable conclusions and consequences can be drawn in terms of the clinical application.

Therefore, in the light of the evidence reported so far and even according to the most recent guidelines dealing with MFI, genetic evaluation of *FSHB* and *FSHR* genes is recommended only for research purposes.^6^

The combination of *FSHR* and *FSHB* polymorphisms might have a much stronger impact than each of them alone.^18^, ^28^, ^61^ Reasonably, as seen above, FSH concentrations and the pharmacogenomic response to the FSH treatment should account for the combined effect of several *FSHB* and *FSHR* polymorphisms, considering an overall possibility of 27 combinations—three genotypes with three polymorphisms: FSHR ‐29 G > A, FSHR 2039 A > G, and FSHB 211 G > T.^33^

The FSH therapy, albeit being a therapy with strong evidence in the field of MFI, is characterized by the absence of a priori predictive markers of response. In fact, in the light of the evidence that only about half of treated patients might respond to such therapy, the identification of subjects who might benefit become pivotal. A recent work from our group^62^ proved the usefulness of a correct a priori identification of patients to treat with FSH. Moreover, this idea is in great line with the new proposal of diagnostic–therapeutic classification of MFI, as recently proposed.^4^

Therefore, integrating genetic evaluation of polymorphisms of *FSHB* and *FSHR* in addition to clinical, biochemical, seminal, and testicular cytological data might be useful in developing a new clinical algorithm in order to predict and even personalize the response to the FSH treatment and adapt the scheme of the treatment (dosage, duration) to maximize the response rate, in particular in patients with testicular disease and normal FSH concentration without evidence of obstruction/sub‐obstruction and with evidence of hypospermatogenesis at the testicular cytological evaluation.

## AUTHOR CONTRIBUTIONS

*Conceptualization*: Andrea Graziani and Giuseppe Grande. *Data curation*: Andrea Graziani, Giuseppe Grande, and Raffele Scafa. *Writing—original draft preparation*: Andrea Graziani, Giuseppe Grande, Raffele Scafa, Riccardo Selice, Maria Santa Rocca, and Cinzia Vinanzi. *Writing—review and editing*: Andrea Garolla and Alberto Ferlin. *Supervision*: Alberto Ferlin. All authors approved the submitted and final versions.

## CONFLICT OF INTEREST STATEMENT

The authors declare no conflicts of interest.
